# Accuracy and safety of ultrasound-guided percutaneous needle core biopsy of renal masses: A single center experience in China

**DOI:** 10.1097/MD.0000000000010178

**Published:** 2018-03-30

**Authors:** Xianding Wang, Yuanhang Lv, Zilin Xu, Muguo Aniu, Yang Qiu, Bing Wei, Xiaohong Li, Qiang Wei, Qiang Dong, Tao Lin

**Affiliations:** a Department of Urology/Institute of Urology, West China Hospital; b West China Medical School; c Department of Pathology, West China Hospital, Sichuan University; d Department of Health Statistics, West China School of Public Health, Sichuan University, Chengdu, Sichuan, China.

**Keywords:** diagnosis, needle core biopsy, renal mass, single center, ultrasound guidance

## Abstract

Our aim is to determine the sufficiency, accuracy, and safety of ultrasound-guided percutaneous needle core biopsy of renal masses in Chinese patients.

Patients who had undergone ultrasound-guided needle core renal mass biopsy from June 2012 to June 2016 at West China Hospital, China were retrospectively reviewed. The information obtained included demographics, mass-related parameters, biopsy indications, technique, complications, pathologic results, and follow-up. Concordance of surgical resection pathology and follow-up data were assessed.

Renal mass biopsies were performed in 106 patients. Thirty-nine (36.8%) were asymptomatic. The male/female ratio was 60/46, with a median age of 49.5 years. Median mass size was 8.1 cm (range 1.8–20). Biopsy was performed through a 16-gauge needle, with median cores of 2 taken (range 1–5). Only one significant biopsy-related complication (hemorrhage requiring transfusion) was encountered. An adequate tissue sample was obtained in 97.2% (103/106) of biopsies. Eighty-seven biopsies (82.1%) showed malignant neoplasms, 16 (15.1%) yielded benignity, and 3 (2.8%) were nondiagnostic. After biopsy, 46 patients (43.4%) underwent surgery. Compared with the subsequent mass resection pathology, the biopsy diagnoses were identical in 43 cases. The accuracy rate of biopsy distinguishing malignant from benign lesions was 99.1%, and the rate for determining tumor histological type (excluding the nondiagnostic biopsies) was 95.1%. The sensitivity and specificity in detecting malignancy were 98.9% and 100%, respectively.

In several situations, there is still a role for biopsy before intervention. Percutaneous needle core biopsy under ultrasonography guidance is highly accurate and safe, and can determine the proper management of undefinable masses.

## 1 Introduction

Multiple treatment modalities, including surgical resection, ablation, neoadjuvant/adjuvant therapy, and active surveillance, are contemporarily available for the management of renal masses.^[1–3]^ This has led to an increasing recognition of the importance of histopathological proof before treatment to characterize radiologically indeterminate renal masses, avoid any unnecessary procedure, and maximize the treatment efficacy.^[4,5]^ However, the current role of percutaneous renal mass biopsy is still controversial, due to concerns regarding its diagnostic accuracy and complications associated with the procedure (e.g., hemorrhage, needle tract seeding).^[6]^ The recent technological advances in the acquisition and interpretation of biopsy specimens have improved the diagnostic yield of renal masses.^[5]^ Several recent studies have shown low complication rate and high diagnostic performance of percutaneous biopsies.^[7]^ In light of these findings, we attempted to report our experience with ultrasound-guided percutaneous needle core biopsies of renal masses in a typical medical setting in China, describe the current indications, accuracy as well as complications of the technique, and evaluate the role of biopsy in the clinical decision making.

## 2 Methods

### 2.1 Patient data

The inclusion criteria were patients who had undergone ultrasound-guided percutaneous needle core biopsy of renal masses between June 2012 and June 2016 at our institution. The data obtained from the medical records included patient demographics, symptoms, renal mass-associated parameters, biopsy indications, technique, complications, histopathological diagnosis, management after biopsy, and follow-up. In detail, the indications for biopsy were due to the need for diagnosis in the following situations: renal mass with atypical/poorly characterized radiological features; radiological diagnosis of unresectable malignancy or mass with distant metastasis; bilateral or multiple solid masses; mass in patients with a previous history of extrarenal malignancy; mass in patients with comorbidity, renal failure, solitary kidney, or old age, in whom surgery is planned; mass that may be caused by infection; small solid mass (<3 cm). Severe biopsy-related complication was defined as the extension of hospital stay, or the need of transfusion, embolization, or surgical intervention.

### 2.2 Biopsy procedure

Written informed consent was obtained from all patients before the biopsy procedure. Antiplatelet agents or oral anticoagulants were suspended for 3 days before the biopsy. Percutaneous biopsy was performed under ultrasonography guidance, usually in the prone position, by administering 1% lidocaine local anesthesia at the entry site of the needle, with a 16-gauge BARD MAX-CORE disposable core biopsy instrument (length of sample notch: 19 mm, penetration depth: 22 mm). After the procedure, patients were observed for at least 6 hours, and repeat imaging was obtained only for patients with hemodynamic instability. Hemorrhage after biopsy was identified by imaging or clinical evaluation.

### 2.3 Pathology

All biopsy samples were reviewed by experienced pathologists with a particular interest in urogenital tumors. Tumor histology was subtyped according to World Health Organization classification 2009.^[8]^ Biopsy was considered adequate if the length of at least one biopsy sample was ≥10 mm, and nondiagnostic if the sample contained only normal renal parenchyma, blood clot, or necrotic tissue (inability to reach a definitive diagnosis). The biopsy results were compared with the pathological diagnoses of available subsequent surgical resection specimens.

### 2.4 Statistical analyses

The diagnostic accuracy of biopsy was calculated by using sensitivity and specificity tests, and comparing with subsequent surgical resection specimens. All statistical analyses were carried out by SAS 9.0 software (SAS Institute, Cary, NC), with *P* < .05 to be statistically significant.

## 3 Results

Ultrasound-guided needle core biopsies of renal masses were performed in 106 patients from June 2012 to June 2016 at our institution. Thirty-nine patients (36.8%) were asymptomatic, and their renal masses were detected as incidental radiological findings for unrelated medical conditions. Common clinical manifestations in symptomatic patients included abdominal/flank discomfort (26.4%), gross hematuria (17.9%), or systemic symptoms (18.9%) such as weight loss, fatigue, anemia, fever, and edema. The male:female ratio was 60:46, with a median age of 49.5 years (range 13–86). Masses were located in the right kidney (49.1%), left (45.3%), both kidneys (3.8%), and the transplanted kidney (1.9%). The median mass size was 8.1 cm (range 1.8–20). On radiological imaging, 72.6% of renal masses were entirely solid, while 27.4% were solid with cystic components. The clinical indications for biopsy are listed in Table 1. The median biopsy cores taken were 2 (range 1–5). An adequate biopsy tissue (the length of at least 1 biopsy sample was ≥10 mm) was obtained in 97.2% (103/106) of biopsies. The 3 insufficient cases were masses with a predominance of cystic components and contained predominantly necrotic or hemorrhagic areas. The biopsy results are detailed in Table 2. Median sizes of the malignant, benign, and nondiagnostic renal masses were 9.0 cm (range 1.8–20), 6.5 cm (range 2–15.8), and 5.6 cm (range 5.2–7.3), respectively. One severe biopsy-related complication was encountered with an overall morbidity rate of 0.9%. This 62-year-old patient with renal mass size of 5.8 cm presented with post-biopsy hemorrhage and required transfusion of packed erythrocytes. There was no death from biopsy, and no cases of pneumothorax, arteriovenous fistula or tumor seeding along the needle tract were observed during the whole follow-up period.

**Table 1:**
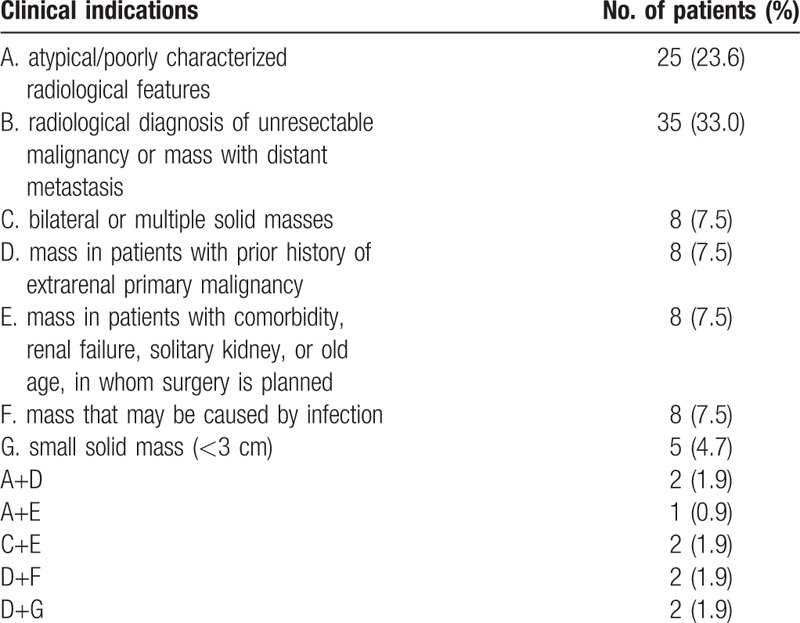
Clinical indications.

**Table 2:**
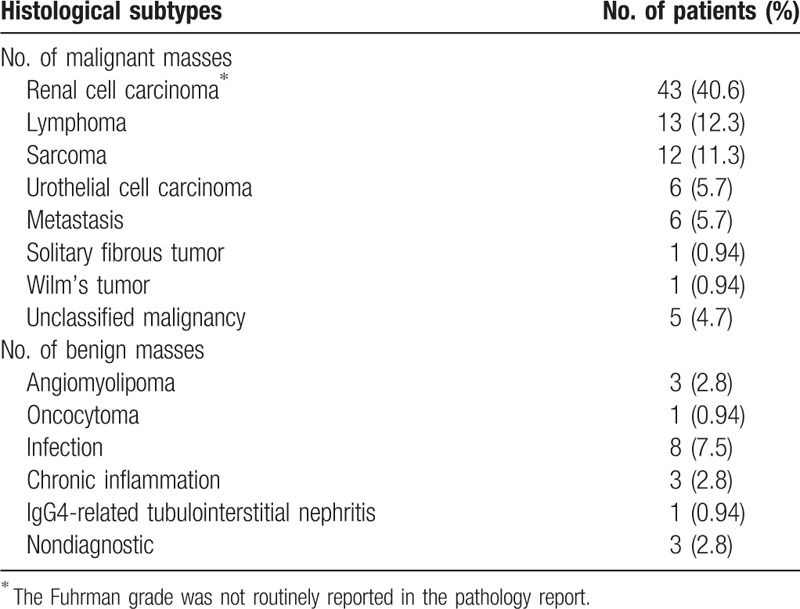
Biopsy result of the renal masses.

The study population was further divided into 2 subgroups according to the mass size (<3 cm vs ≥3 cm). For determining the histological diagnosis, sufficient tissue sample was obtained for evaluation in 11/12 of the mass size <3 cm subgroup and 92/94 of the mass size ≥3 cm subgroup. The biopsy diagnostic rates in the <3 cm and ≥3 cm subgroups were 100% and 96.8%, respectively. After biopsy, 6/12 patients in the <3 cm subgroup and 40/94 in the ≥3 cm subgroup underwent surgical resection. The biopsy diagnosis was compared with the subsequent mass resection pathology, and diagnoses were identical in 43 cases: 5/6 in the <3 cm subgroup and 38/40 in the ≥3 cm subgroup. Histological subtype was changed from nondiagnostic to multilocular cystic renal cell carcinoma in one case, and from unclassified malignancy to primitive neuroectodermal tumor and carcinoma of the collecting ducts of Bellini, respectively, in 2 cases. As the patient sample size in the <3 cm subgroup was relatively small (12 cases), the subgroup analysis on the mass size to determine the diagnostic accuracy and clinical benefit was precluded.

Among 87 patients with a biopsy diagnosis of malignancies, 42 underwent radical, partial, or palliative nephrectomy. The remaining patients were medically unfit, or refusal of surgery. Active surveillance was the mode of treatment in nonmalignant masses with the exception of 3 patients with infection (renal abscess drainage) and 1 patient with nondiagnostic. For this nondiagnostic case, the patient was a 39-year-old male with growth of renal mass during follow-up and underwent surgical resection; a diagnosis of multilocular cystic renal cell carcinoma was made in the surgical specimen. In the remaining cases, there has been no significant change in the renal mass size with regular follow-up. Therefore, this confirmed a 99.1% biopsy accuracy rate of distinguishing malignant from benign lesions and a 95.1% rate for determining tumor histological type (excluding the nondiagnostic biopsies). The sensitivity of the percutaneous needle biopsy in detecting malignancy was 98.9% and specificity 100%.

## 4 Discussion

Historically, surgical resection was the mainstay for the management of renal masses without the need of biopsy before resection.^[5]^ However, pretreatment characterization of renal mass pathology would prevent unnecessary procedures and assist the urologists to choose the most suitable therapy. Barwari et al^[9]^ surveyed the members of the Endourological Society on the use of renal mass biopsy in the daily practice. Seventy-three percent of responders indicated performing biopsy “never” or “rarely” compared with 9% performing in 25% to 100% of masses; the main indications to perform biopsy were masses in solitary/transplanted kidney or metastatic disease. Lack of influence on clinical management and risk of false negative results were the main reasons not to perform biopsies. Unlike other studies that are confined to only 1 or 2 indications to perform biopsy,^[12,15]^ our study included almost all established indications (see details in Section 3) that have been derived from previous literature and a wealth of our clinical experience,^[10]^ and faithfully revealed the recent epidemiological situation of renal masses in China where medical resources are limited. Furthermore, our current indications for biopsy have been shifted from traditional (i.e., radiological diagnosis of unresectable malignancy or mass with distant metastasis) to some new, emerging indications including small solid masses (<3 cm), and masses in the elderly or frail patients, in which less invasive therapy strategies may be more desirable.^[11]^ A possible explanation is that currently with the extensive application of the imaging modalities such as computed tomography and ultrasonography in the general clinical practice, 70% of renal masses are detected incidentally and presumably likely to be at a small size and a low stage posing therapeutic dilemmas.^[12]^

A significant number of patients who proceed directly to aggressive surgical resection, but who truly have a nonmalignant or certain indolent malignant lesion, are potentially undergoing an unnecessary surgical procedure.^[13]^ Meanwhile, in patients with a large locally advanced or metastatic renal malignancy, histological diagnosis before surgery is important in guiding oncologists to choose a suitable chemotherapy and/or radiotherapy regimen.^[14]^ Furthermore, intravenous contrast agent is contraindicated in patients with chronic renal failure who are at risk of developing bilateral, multiple renal cell carcinomas, and these renal masses may be incompletely/only partially evaluated radiologically. Renal mass biopsy, therefore, has become a powerful tool to clarify clinico-radiological dilemmas, and plays an important role in guiding individualized patient management, particularly by stratifying patients for active surveillance or surgical resection. In the present study, an adequate biopsy tissue was strictly defined as the length of at least one biopsy sample was ≥10 mm, and was obtained in 97.2% of our patients. The insufficient cases were masses with a predominance of cystic components and contained predominantly necrotic or hemorrhagic areas. Approximately 79% of the nonmalignant masses were actively surveyed as compared with 10% of the malignant ones. Although our study did not investigate the changes in the paradigm of care after biopsy, other studies have shown that biopsy alters clinical management in 41% to 61% of patients in whom a biopsy is performed.^[15]^ For example, a pathological diagnosis of an incidentally detected small low-grade neoplasm would support using a minimally invasive technique (e.g., ablation, nephron-sparing surgery), or even active surveillance in some elderly or unfit patients. Finally, when active surveillance is chosen, delayed surgical resection can also be reserved for masses that exhibit a quick growth during follow-up, and there is no evidence that biopsy may complicate the subsequent surgery.

Our study demonstrated that an adequate tissue can be obtained in 97.2% of biopsies, and major clinical complications from biopsy are rare (0.9%). Although concern for needle tract seeding is a potential complication of renal mass biopsy, the true incidence is actually negligible with only a few case reports in the past decades.^[16]^ Marconi et al^[17]^ performed a meta-analysis of the literature to determine the diagnostic performance and safety of renal tumor biopsy. The overall median diagnostic rate of biopsy was 92%, with a sensitivity and specificity of 99.1% and 99.7%, respectively. A very low rate of Clavien ≥2 complications was reported. Due to advances in pathological techniques including the molecular and genetic tests, false-positive results are exceedingly rare. False-negative and nondiagnostic cases, on the other hand, can be occasionally encountered primarily when there is a predominance of necrotic/hemorrhagic areas.^[18]^ False-negative may be further improved by a combination of core biopsy and fine-needle aspiration.^[19]^ The absence of malignancy from a biopsy does not necessarily confirm benignity and should be under close follow-up especially in the nondiagnostic cases. For renal masses <4 cm, larger tumor size and a solid nature predicted a successful biopsy on multivariate analysis;^[20]^ however, when the mass is large in size (≥4 cm), the accuracy rate of core biopsy is not increased. Menogue et al found that increasing the number of cores taken improved the diagnostic success on univariate analysis. However, on multivariable analysis, when adjusted for the amount of tissue available for diagnosis, the number of cores was no longer a significant predictor of success.^[21]^ Using a controlled, ex-vivo biopsy technique, Breda et al prospectively compared the accuracy of 14-, 18- and 20-gauge core needles, and found that the biopsy histology correlated with the final pathology in 94% of cases with the 14-gauge, 97% with the 18-gauge and 81% with the 20-gauge needles.^[22]^ Therefore, a minimum of an 18-gauge biopsy needle may be the most accurate in determining the histological diagnosis. This was the case in our center where biopsies were performed with a 16-gauge needle. The proven safety of larger needles together with our clinical experience prompted us to use a 16-gauge needle in an attempt to get a high diagnostic rate. Leveridge et al^[20]^ examined the role of repeat biopsy, which was performed in 12 of 67 nondiagnostic cases, and a diagnosis was possible in 10 (83.3%), making repeat biopsy an appealing option.

There are several potential limitations affecting the validity of our findings. First, one obvious and major limitation is the retrospective nature and some important data are unavailable. Second, 56.6% of our patients did not undergo subsequent surgical resection; thus, there was a lack of surgical pathological confirmation for the accuracy of these biopsies. Third, the series examining the results of biopsy apply to selected patients. Not all renal masses meeting the biopsy indications were referred for biopsy; instead, some patients proceeded directly to therapy, resulting in a selection bias. Fourth, we did not perform a repeat biopsy in nondiagnostic cases. Repeat biopsy may be warranted in cases with suspicious clinical features and inadequate biopsy findings in future studies. Fifth, we are unable to comment on the safety and efficacy of different imaging guidance methods (e.g., computed tomography vs ultrasound) because all our biopsies were performed under ultrasonography.

In conclusion, although imaging is the fundamental tool in the evaluation of renal masses, in several specific situations, there is still a role for imaging-guided biopsy before intervention. Percutaneous needle core biopsy under ultrasonography guidance is highly accurate and safe. The findings from biopsy can significantly determine the proper management of suspicious and undefinable masses, and could potentially avoid surgical resection and its downstream complications.

## Author contributions

**Conceptualization:** X.D. Wang.

**Data curation:** M.G. Aniu, X.D. Wang, Y. Qiu, Y.H. Lv.

**Formal analysis:** B. Wei, M.G. Aniu, X.D. Wang, Y.H. Lv.

**Funding acquisition:** Q. Dong.

**Investigation:** B. Wei, Y.H. Lv.

**Methodology:** X.H. Li, Z.L. Xu.

**Project administration:** Q. Dong, X.H. Li.

**Resources:** Q. Wei.

**Software:** X.H. Li, Z.L. Xu, Tao Lin.

**Validation:** Y. Qiu.

**Visualization:** Q. Dong.

**Writing – original draft:** X.D. Wang, Y. Qiu.

**Writing – review & editing:** T. Lin.
